# Conditional ablation of HDAC3 in islet beta cells results in glucose intolerance and enhanced susceptibility to STZ-induced diabetes

**DOI:** 10.18632/oncotarget.11295

**Published:** 2016-08-15

**Authors:** Wen-Bin Chen, Ling Gao, Jie Wang, Yan-Gang Wang, Zheng Dong, Jiajun Zhao, Qing-Sheng Mi, Li Zhou

**Affiliations:** ^1^ Henry Ford Immunology Program, Henry Ford Health System, Detroit, MI, USA; ^2^ Central Laboratory, Shandong Provincial Hospital Affiliated to Shandong University, Jinan, China; ^3^ Department of Dermatology, Henry Ford Health System, Detroit, MI, USA; ^4^ Department of Endocrinology, Affiliated Hospital of Qingdao University, Qingdao, China; ^5^ Department of Cellular Biology and Anatomy, Augusta University, GA, USA; ^6^ Department of Endocrinology, Shandong Provincial Hospital affiliated to Shandong University, Jinan, China; ^7^ Department of Internal Medicine, Henry Ford Health System, Detroit, MI, USA

**Keywords:** HDAC3, knockout, autoimmune diabetes, glucose tolerance, insulin, Pathology Section

## Abstract

Histone deacetylases (HDACs) are enzymes that regulate gene expression by modifying chromatin structure through removal of acetyl groups from target histones or non-histone proteins. Previous *in vitro* studies suggest that HDACs may be novel pharmacological targets in immune-mediated islet β-cell destruction. However, the role of specific HDAC in islet β-cell development and function remain unclear. Here, we generated a conditional islet β-cells specific HDAC3 deletion mouse model to determine the consequences of HDAC3 depletion on islet β-cell differentiation, maintenance and function. Islet morphology, insulin secretion, glucose tolerance, and multiple low-dose streptozotocin (STZ)-induced diabetes incidence were evaluated and compared between HDAC3 knockout and wild type littermate controls. Mice with β-cell-specific HDAC3 deletion displayed decreased pancreatic insulin content, disrupted glucose-stimulated insulin secretion, with intermittent spontaneous diabetes and dramatically enhanced susceptibility to STZ-induced diabetes. Furthermore, islet β-cell line, MIN6 cells with siRNA-mediated HDAC3 silence, showed decreased insulin gene transcription, which was mediated, at least partially, through the upregulation of suppressors of cytokine signaling 3 (SOCS3). These results indicate the critical role of HDAC3 in normal β-cell differentiation, maintenance and function.

## INTRODUCTION

Diabetes mellitus is a group of multi-factorial metabolic diseases characterized by absolute or relative deficiencies in insulin production. Molecular mechanisms affecting pancreatic β-cell differentiation, proliferation, survival, and function are fundamental for understanding the pathogenesis of both type 1 and type 2 diabetes. Preserving a functional β-cell mass and promoting the β-cell differentiation, proliferation and function are therefore the primary targets of novel treatments aimed at curing and preventing diabetes mellitus.

Histone deacetylases (HDACs) are enzymatic components of large multi-protein complexes that remove acetyl groups from lysine side chains of histones and other proteins. They contribute to epigenetic programming and regulation of gene expression during development and throughout life. Gene transcription is modulated by acetylation and deacetylation of histones. Acetylation of histones generally contributes to the formation of a transcriptionally competent environment by relaxing the chromatin structure, allowing transcription factors to access the target DNA. In contrast, histone deacetylation compacts chromatin and leads to transcriptional repression [1] Recent studies also suggested that HDACs regulate the activity of a wide range of non-histone proteins [2], thus protein acetylation has a big impact in posttranslational regulation of proteins.

Mammalian HDACs are classified into four groups on the basis of their structure and function. HDAC3 is part of class I, which also includes HDAC1, 2, and 8 [3]. Although class I HDACs are broadly expressed and structurally related, they have distinct developmental roles [4, 5]. All HDACs were found expressed in rat β-cell line, INS-1 cells, and rat islets, implying their potential roles in islet β-cell development and function [6]. Different HDACs participate in specific multiprotein repressive transcriptional complexes, which could potentially determine, at least in part, their unique functions.

HDAC3 is a component of the nuclear receptor co-repressor complexes that contain N-CoR and SMRT [7]. Using cell specific HDAC3 deletion mouse models, previous studies have revealed the critical roles of HDAC3 in cardiac functional maintenance and myocardial energy metabolism [8], liver metabolic homeostasis [9, 10], and in bone formation and bone marrow adipocyte differentiation [11]. Previous studies suggest that HDACs may be novel pharmacological targets in immune-mediated β-cell destruction leading to type-1 diabetes. HDAC inhibitors (HDACi) reduce cytokine induced β-cell toxicity and restore insulin secretion both *in vitro* and *in vivo* [6, 12–14], in which class I HDACs, especially HDAC1 and HDAC3 may play essential role [13, 15]. However, the specific role of HDAC3 in islet β-cell development, differentiation, maintenance and function remain unclear. Here, we made a conditional islet β-cells specific HDAC3 deletion mouse model to determine the consequences of HDAC3 depletion on islet β-cell differentiation, maintenance and function.

## RESULTS

### Deletion of HDAC3 in islet β-cells in mice

**Figure 1:**
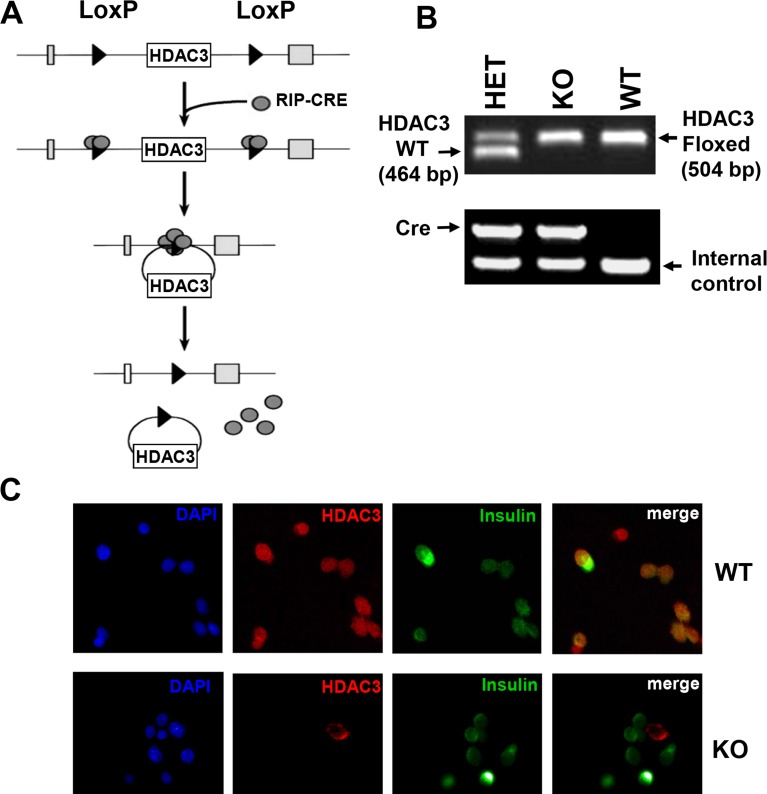
Generation of β-cell-specific HDAC3 knockout mice **A.** The floxed and null alleles of HDAC3 as a result of recombination between LoxP sites. **B.** PCR results of genotyping from tail biopsies of RIP-Cre^−^ HDAC3^fl/fl^ (WT), RIP-Cre^+^ HDAC3^fl/+^ (HET) and RIP-Cre^+^ HDAC3^fl/fl^ (KO) mice. **C.** Dispersed islet cells from WT and KO mice were immunostained for HDAC3 (red) and insulin (green). Yellow (merged) indicates coexpression of HDAC3 and insulin. (Scale bar, 20 μm).

β-cell specific HDAC3 knockout mice (RIP-Cre^+^.HDAC3^fl/fl^) were generated by mating mice expressing the Cre recombinase gene under the control of rat insulin 2 gene promoter (RIP-Cre^+^) with HDAC3^flox/flox^ mice [10] to obtain RIP-Cre^+^HDAC3^fl/+^ mice, which were then crossed with HDAC3^fl/fl^ to get RIP-Cre^+^.HDAC3^fl/fl^ knockout (KO) mice (Figure 1A). Littermates carrying HDAC3^fl/fl^, without cre expression (RIP-Cre^−^ HDAC3^fl/fl^) were used as wild type (WT) controls. Since RIP-Cre transgenic mice have been suggested to show glucose intolerance, heterozygous RIP-Cre^+^.HDAC3^fl/+^ (HET) mice were also used as control for glucose tolerance experiment. HDAC3^flox/flox^ and RIP-Cre alleles were identified by PCR with related primers (Table S1) (Figure 1B). To confirm HDAC3 deletion in islet β-cells from RIP-Cre^+^ HDAC3^fl/fl^ (HDAC3 KO) mice, dispersed islet cells from these mice and WT control littermates (RIP-Cre^−^ HDAC3^fl/fl^) were co-stained for HDAC3 and insulin. As shown in Figure 1C, insulin and HDAC3 co-expression was observed in dispersed WT β-cells, while none of the insulin expressing β-cells express HDAC3 in dispersed β-cells from HDAC3 KO mice, indicating the efficient β-cells specific HDAC3 deletion in these mice (Figure 1C).

### Spontaneous glucose metabolism features of RIP-Cre^+^ HDAC3^fl/fl^ mice

**Figure 2:**
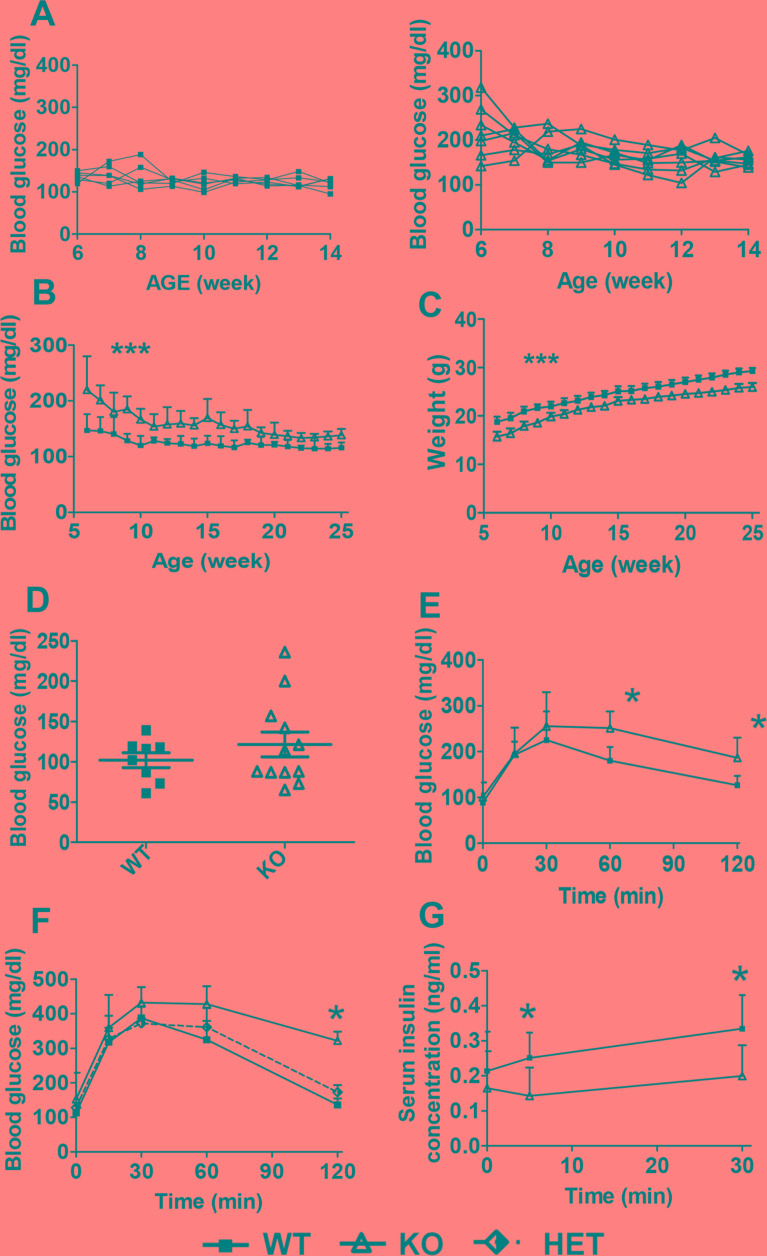
β-cell-specific deletion of HDAC3 causes higher blood glucose level, glucose intolerance and impaired insulin secretion in mice **A.** Random blood glucose levels were monitored in WT (left) and KO (right) mice, each line represents an individual mouse. *n* = 6-7 male mice/group. **B.** The summary of non-fasting blood glucose levels and **C.** body weight monitored from 6- until 25-week of age. *n* = 6-7 male mice per group. The overall difference between KO and WT group was analyzed by ANOVA. **D.** Fasting blood glucose level was measured in 8- to 12-week-old KO and WT male mice. *n* = 8-12 male mice/group. Glucose tolerance test, 1g/kg body weight **E.** or 2g/kg body weight **F.**, was performed on 8- to 10-week-old KO and WT mice. *n* = 4-6 mice/group. **G.** Plasma insulin levels during glucose tolerance test at the dose of 2 g/kg body weight. *n* = 5-6 male mice/group. Data are presented as means ± SD. (*p < 0.05, ***p < 0.001).

Throughout the monitoring time (5 to 25 weeks of age), HDAC3 KO mice showed a moderately decreased body weight compared with the WT control littermates (Figure 2C). Meanwhile, HDAC3 KO mice displayed steady higher blood glucose levels, starting at 6 weeks old, especially before 10 weeks old (Figure 2A and 2B). Fasting blood glucose levels were measured and compared between HDAC3 KO and WT control mice. All WT mice showed normal fasting glucose levels, while 2 out of 12 HDAC3 KO mice showed elevated fasting glucose levels. Nevertheless, the overall fasting blood glucose level in HDAC3 KO group showed no significant difference comparing to that of WT control group (Figure 2D).

### Glucose intolerance and impaired glucose-stimulated insulin secretion

Glucose tolerance tests (GTT) was performed to assess the body's response to glucose, including insulin secretion capacity. Male mice were i.p. injected with glucose of 1 or 2 g/kg of body weight, and impaired glucose clearance was observed in RIP-Cre^+^HDAC3^fl/fl^ mice at both doses compared to WT controls (Figure 2E and 2F). Glucose tolerance in RIP-Cre^+^HDAC3^fl/+^ mice was indistinguishable from RIP-Cre^−^HDAC3^flox/flox^, indicating that glucose homeostasis was not affected by HDAC3 heterozygosity or RIP-Cre expression in β-cells. Insulin secretion following the glucose administration was measured. At 5 and 30 min post glucose injection, HDAC3 KO mice showed impaired insulin secretion compared to WT controls which is consistent with increased glucose levels in the same animals (Figure 2F).

### Increased susceptibility to multiple low-dose streptozotocin (MLD-STZ)-induced diabetes

**Figure 3:**
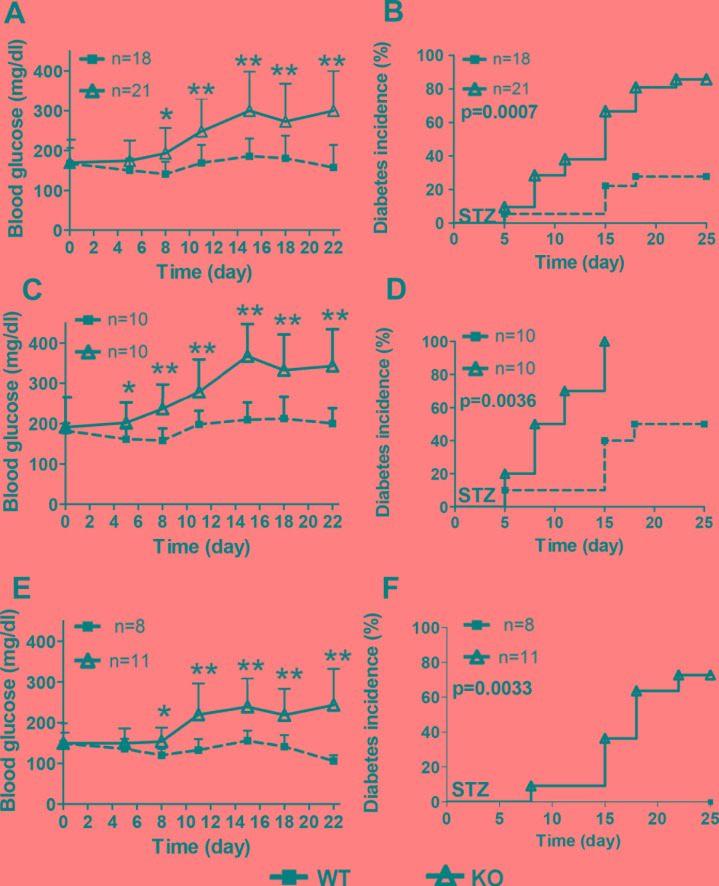
RIP-Cre.HDAC3^fl/fl^ mice are more susceptible to multiple low-dose STZ-induced (MLD-STZ) diabetes Blood glucose levels throughout the MLD-STZ experiment were monitored and compared between KO and WT for all mice **A.**, male mice **C.** or female mice **E.**, respectively. Diabetes incidence was monitored and compared between KO and WT mice in all mice **B.**, male mice **D.** or female mice **F.**, respectively. Data are presented as means ± SD. (*p < 0.05, **p < 0.01).

**Figure 4:**
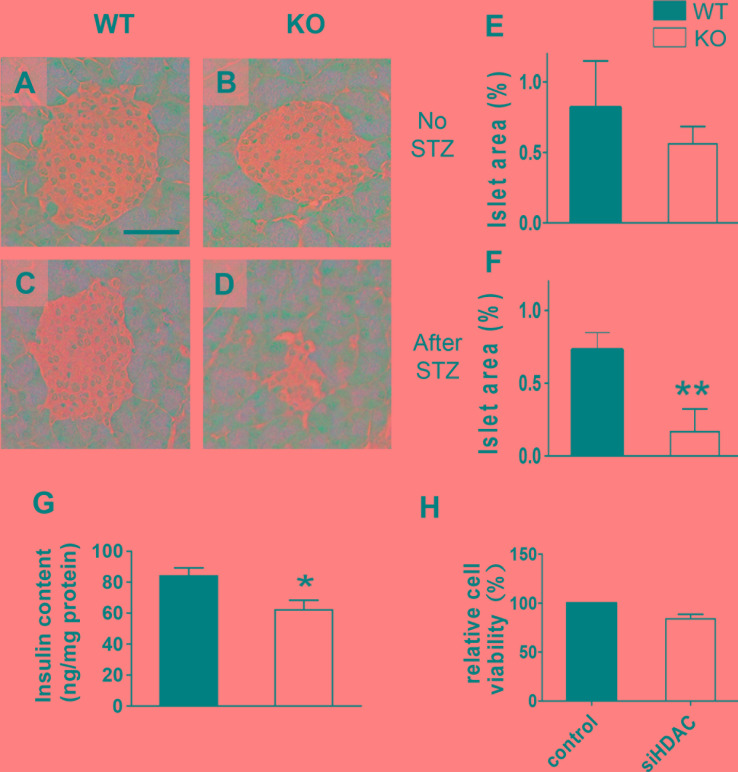
Histopathological analysis of islets of β-cell-specific HDAC3 KO mice Pancreatic sections were stained with hematoxylin and eosin from WT and KO mice without STZ treatment **A.** and **B.** or 25 days after STZ injection **C.** and **D.**, scale bar, 100 μm.). Islet area relative to total pancreas was evaluated and compared between KO and WT mice of untreated **E.**, or 25 days after STZ treatment **F.**. *n* = 10-13 mice per group. Pancreatic insulin content was measure and compared between 8-12-week-old untreated KO and WT mice. *n* = 4-5 mice per group **G.**. The relative viability of Min6 cells transfected with non-targeting sequence siRNA or HDAC3-specific siRNA was compared using MTT assay **H.**. (*p < 0.05, **p < 0.01).

To study the sensitivity of β-cell to toxicity and inflammation induced death, we set out to induce diabetes by MLD-STZ injections to animals. Blood glucose levels of HDAC3 KO mice showed dramatic increase compared to WT controls from day 8 till the end of the experiment (Figure 3A). When separating male and female mice into individual groups, the increased blood glucose levels in HDAC3 KO mice remained in both groups (Figure 3C and 3E). Consistent with different blood glucose levels, 60% of the HDAC3 KO mice, while only 20% from WT controls developed diabetes by day15. By day25, more than 80% of HDAC3 KO mice, while only about 27% from WT controls developed diabetes (Figure 4B). Both male and female KO mice showed significant increased diabetes incidence compared to WT controls, respectively (Figure 3D and 3F).

### Decreased β-Cells and insulin content

Relative lower body weight, hyperglycemia, glucose intolerance, and dramatically increased sensitivity to STZ treatment in HDAC3 KO mice strongly suggest a developmental and/or functional defect in pancreatic islet β-cells. Histological analysis of pancreatic sections revealed no obvious malformation of islet structure, but a trend of decreased overall islet area in HDAC3 KO mice was identified (Figure 4A, 4B and 4E *p* = 0.07). Meanwhile, HDAC3 KO mice showed significantly decreased pancreatic insulin content, in consistent with the trend of decreased islet β-cell mass (Figure 4G). To further evaluate the potential role of HDAC3 in β-cell viability, MTT assay was performed to compare the viability of Min-6 cells with HDAC3 silence or control, and no significant difference was identified (Figure 4H). Nevertheless, after STZ treatment, the histological islet β-cell mass decreased dramatically in HDAC3 KO mice, in consistent with their dramatically increased blood glucose level and diabetes incidence (Figure 4C, 4D and 4F; Figure 3). Taken together, these results strongly suggest that in addition to the potential involvement in islet β-cell development, HDAC3 also plays important roles in β-cell insulin secretion and homeostasis.

### Direct regulation of SOCS3 by HDAC3 in islet β-cells contributes to insulin production

**Figure 5:**
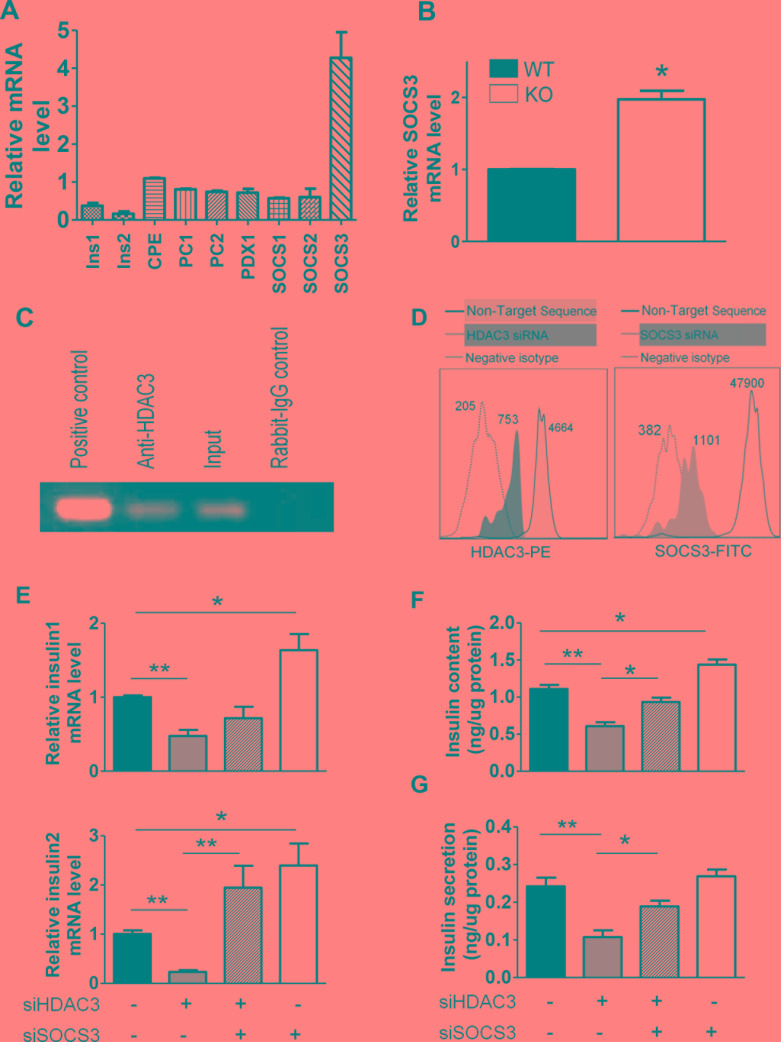
SOCS3 upregulation by HDAC3 depletion in islet β-cells contributes to insulin production defect **A.** Real-time PCR analysis of insulin related gene mRNA changes in HDAC3 silenced Min6 cells. **B.** Real-time PCR analysis of changed SOCS3 mRNA levels in HDAC3 KO and WT control mice. **C.** CHIP assays were performed with Min6 cells. Chromatin was immunoprecipitated with anti-HDAC3 or rabbit IgG control antibody, and precipitated DNA was used as a template to detect the SOCS3 promoter. Nonimmunoprecipitated sample served as an input control. **D.** Flow cytometry analysis of Min6 cells with HDAC3 or SOCS3 silencing. **E.** Real-time PCR analysis of Insulin1 and Insulin2 mRNA levels in different siRNA transfected Min6 cells. Insulin content **F.** and glucose stimulated insulin secretion **F.** of Min6 cells with silenced HDAC3, SOCS3 or both. Data are presented as means ± SD. (*p < 0.05, **p < 0.01).

To investigate the potential molecular mechanisms involved in the insulin secretion defect of HDAC3 depleted β-cells, Min6 cells were transfected with HDAC3 siRNA. The expression of classical insulin relevant genes as well as genes involved in insulin secretory pathways and potentially regulated by HDAC3 was evaluated (Figure 5A). The mRNA level of SOCS3 was significantly increased in both HDAC3 silenced Min6 cells and islets from RIP-Cre^+^ HDAC3^fl/fl^ mice (Figure 5A & 5B). To explore the potential regulatory role of HDAC3 on SOCS3, CHIP assay was performed to detect the potential binding of HDAC3 to the promoter of SOCS3. Immunoprecipitation of HDAC3 with anti-HDAC3 was able to robustly pull down the promoter of SOCS3 gene compared with rabbit IgG control antibody (Figure 5C). This result shows the direct binding of HDAC3 to SOCS3 gene, indicating the direct regulatory role of HDAC3 on SOCS3 expression. To further confirm the regulatory role of HDAC3 and the involvement of SOCS3 in HDAC3 deficiency induced β-cell dysfunction, Min6 cells were co-transfected with siRNAs against HDAC3 and SOCS3. Seventy-two hours after transfection, a flow cytometry analysis was performed to confirm the silence efficiency (Figure 5D). As expected, HDAC3 silence interrupted the transcription of both insulin1 and insulin2. In contrast, SOCS3 silence increased insulin transcription in Min6 cells. Nevertheless, HDAC3 and SOCS3 co-silence reversed the inhibitory effect of HDAC3 silence on insulin transcription, but only insulin2 mRNA change reach the statistical significant level (Figure 5E). Furthermore, in agreement with the insulin mRNA changes, SOCS3 silence elevate the insulin content, while the co-silence of SOCS3 and HDAC3 rescue the decreased insulin content and secretion induced by HDAC3 silence (Figure 5F & 5G).

## DISCUSSION

HDAC3 is essential for embryonic development [4, 5]. It associates with numerous transcription factors, co-repressors, class II HDACs [7, 16, 17], and plays critical roles in multiple organ development, metabolic homeostasis and function [8, 11]. In the current study, we made mouse model with conditional deletion of HDAC3 in islet β-cells to investigate the roles of HDAC3 in islet β-cell development and function [18, 19]. Mice with β-cell-specific HDAC3 deletion displayed decreased insulin content, disrupted glucose-stimulated insulin secretion and tolerance, and dramatically enhanced susceptibility to low dose STZ induced diabetes. These data strongly indicate the important role of HDAC3 in islet β-cell insulin secretion capacity and vulnerability of β-cells to toxic and inflammatory attack in addition to its potential involvement in β-cell differentiation. Furthermore, Min6 cells with siRNA mediated HDAC3 silence showed decreased insulin gene transcription, which was mediated, at least partially, through up-regulation of SOCS3. These results not only confirm the critical role of HDAC3 in β-cell insulin secretion function, but also illuminate the potential molecular mechanisms of HDAC3 mediated islet β-cell functional regulation. Together, studies from both *in vivo* and *in vitro* models indicate that HDAC3 is essential for proper islet β-cell differentiation, function and maintenance.

The roles of HDACs in pancreatic developmental regulation remain largely unknown. Class I and class II HDACs are expressed in the embryonic pancreas. The pancreatic expression of HDACs are developmentally regulated, with significantly decreased most class I and II HDAC expression upon differentiation [20], consistent with the regulated HDACs expression during osteogenesis and adipogenesis [21, 22]. In contrast to other HDACs, HDAC3 expression was dramatically up-regulated in adult pancreas compared to different embryonic stages, suggesting the potential role of HDAC3 in not only pancreatic development, cell differentiation but also their function and maintenance [20].

Treatment of rat embryonic explants with HDAC inhibitors (HDACi) *ex vivo* promoted the NGN3 pro-endocrine lineage development, leading to an increased pool of endocrine progenitors. However, different HDACi showed opposing effects on the endocrine β/δ lineage differentiation, in which preferential Class I HDACi VPA/MS275 induced a dramatic decrease in Pax4 and insulin expression, while the Class I and II HDACi TSA/NaB enhanced Pax4 and insulin expression, suggesting the specific roles of individual HDACs in the development and differentiation of pancreatic β-cells [20]. Consistent with these results, mice with β-cell specific HDAC3 deletion in our study showed decreased pancreatic insulin content, trend of decreased β-cell mass, moderate hyperglycemia, which could result, at least partially, from the interrupted β-cell linage differentiation induced by HDAC3 deletion.

HDAC3 is among the β-cell expressed HDACs down-regulated by inflammatory cytokine treatment of both primary β-cells and β-cell lines [6, 23]. Pan-HDACi ITF2357 treatment dose-dependently reversed cytokine induced β-cell death and the impairment of insulin secretion in INS-1 cells and primary rat β-cells, respectively; whereas ITF2357 itself had no effect on primary rat β-cell insulin secretion and survival, except for high dose of ITF2357 showing a trend of promoting effects on INS-1 apoptosis [6]. Using individual HDAC deleted INS-1 cells or primary rat β-cells, a more recent study further confirmed that HDAC1 and HDAC3 deletion can partially rescue cytokine-induced β-cell apoptosis. Nevertheless, the individual HDAC deletion itself had no effects on β-cell survival [15]. Multiple low dose-streptozotocin (MLD-STZ) induces diabetes through primary β-cell toxicity plus secondary local inflammation [24]. In the current study, mice with β-cell-specific HDAC3 deletion displayed significantly enhanced susceptibility to MLD-STZ treatment compared to littermate controls, which could result from either interrupted primary β-cell differentiation and function, or the increased sensitivity of β-cells to the toxicity of STZ and inflammatory factors, or both. This result is inconsistent with aforementioned protective role of HDACis in inflammatory cytokine mediated β-cell apoptosis and functional defects. Comparing these studies with our model system, there are a few possible explanations for the apparent discrepancies: first, Cre-mediated recombination of HDAC3 in β-progenitor cells is a permanent event and the phenotypes may result from HDAC3 deletion-mediated β-cell development, differentiation as well as mature β-cell function and/or maintenance defects. In contrast, small molecule HDACis have short half-life in serum or culture medium and might only transiently change chromatin structure and gene expression in normally developed β-cells in relatively short-term assays. Second, HDACs participate in multiprotein transcriptional complexes. Genetic deletion of an HDAC perturbs the complexes in which it would normally be associated, whereas inhibitors are believed to block enzymatic activity without necessarily disrupting the repressive complex. Furthermore, striking difference exist in etiopathogenesis of β-cell destructions induced by cytokine treatment *versus* MLD-STZ which include primary β-cell toxicity from STZ plus secondary local inflammation [24].

SOCS are intracellular proteins that control signals from cytokine receptors and are essential for immune physiology. SOCS proteins are induced by cytokines and act to inhibit cytokine signal transduction. SOCS3 has been reported to protect against cytokine mediated β-cell destruction by suppressing IFN-γ and IL-1β signaling *in vitro* [25, 26]. In mice, however, β-cell specific overexpression of SOCS3 failed to protect against MLD-STZ induced diabetes [27]. Leptin-mediated SOCS3 up-regulation transcriptionally inhibits preproinsulin gene expression in β-cells [28]. In addition to the impact of SOCS3 on β-cell insulin production, SOCS3 inhibits GH-mediated β-cell proliferation, and mice with SOCS3 overexpression showed over 30% β-cell mass reduction [29]. However, mice with haploinsufficiency of SOCS3 showed enhanced leptin sensitivity and were protected against the development of diet-induced obesity and associated metabolic complications [30]. Furthermore, elevated SOCS3 expression suppressed insulin signaling through inhibiting the phosphorylation of insulin receptor (IR) and insulin receptor substrates (IRS), while promoting the degradation of IRS1 and IRS2 [31–33] These results clearly demonstrate the essential role of SOCS3 in balancing islet β-cell mass, function and even insulin sensitivity. In the current study, siRNA silencing of HDAC3 dramatically upregulate SOCS3 expression in Min6 cells, in concord with interrupted insulin production, while SOCS3 silencing partially reverse the down regulation of insulin transcription. These results indicate the critical involvement of SOCS3 in HDAC3 deletion induced β-cell functional defects. Nevertheless, we cannot rule out the possibilities of the involvement of additional genes whose activation or expression is under the control of HDAC3.

HDAC3-containing N-CoR/SMRT complexes bind preferentially to hypoacetylated histones [34, 35]. However, class I HDACs not only remove acetyl groups from histones tails but can also deacetylate transcription factors and other chromatin-associated proteins. Thus, class I HDACs can affect gene transcription at different levels [36]. Recent studies have shown the evidence of HDAC mediated SOCS3 expression regulation: HDACi treatment can promote the SOCS1 and SOCS3 expression in human colorectal cancer and human leukemia through inducing the hyperacetylation of histones associated with the SOCS3 promoters [37, 38], which further suppress Janus kinase/signal transducers and activators of transcription (JAK/STAT) signaling involved in the oncogenesis of different cancers [39]. Thus, these results link SOCS proteins with the anti-tumor functions of HDACis, which further support our findings that SOCS3 is one of the major molecules involved in HDAC3-mediated islet β-cell differentiation, and function. In addition, our CHIP data suggested the direct regulatory role of HDAC3 on SOCS3 expression. These data combined with previous identified hyper acetylated histone-associated SOCS3 expression regulation indicate the existence of complex multi-pathways in HDAC mediated SOCS protein regulation including at least the conventional Histone deacetylation-mediated and the direct regulation pathway possibly recruited by SOCS3 regulatory transcription factors. The detailed molecular mechanisms of SOCS3-dependent and -independent pathways of HDAC3 related islet β-cell development, differentiation and functional regulation will be further investigated in future studies.

In summary, using the islet β-cell specific HDAC3 deletion mouse model combined with HDAC3 silenced Min6 cell line, our results indicate the critical role of HDAC3 in islet β-cell differentiation, function and maintenance, and the role of SOCS3 in HDAC3 deletion induced β-cell dysfunction. These results not only provide further insight into the epigenetic mechanisms of β-cell development and functional regulation, but also emphasize the importance of understanding the role and mechanisms of individual HDACs in β-cell regulation, for future HDAC related clinical applications with increased specificity and minimized toxicity in the treatment and prevention of diabetes mellitus and other diseases.

## MATERIALS AND METHODS

### Islet isolation, dispersion, immunofluorescence staining, and pancreas histological and immunofluorescence analysis

Mouse islets were isolated by type V collagenase (Sigma Chemicals, St Louis, MO, USA) digestion of pancreas. Isolated islets were exposed to 0.25% trypsin-EDTA at 37°C for 10 min, followed by gently pipetting for 1-2 min to dissociate into single cells. Dispersed islets cells were fixed in 4% (vol./vol.) paraformaldehyde for 10 min, permeabilized with 0.25% (vol./vol.) Triton X-100 for 10 min and blocked overnight in 1% (wt/vol.) BSA at 4°C. Cells were then labeled overnight at 4°C with polyclonal anti-HDAC3 (Abcam, Cambridge, MA, USA) and monoclonal anti-insulin (Linco, Research, Inc., St Louis, MO, USA) antibodies. Cells were then stained at room temperature in dark for 60 min with PE-conjugated goat anti-rabbit, FITC-conjugated goat anti-mouse secondary antibodies (eBioscience, San Diego, CA, USA) respectively. Cells were washed and mounted on glass slides which were viewed on a microscope (Leica Microsystems, Wetzlar, Germany).

Pancreases obtained from 8- to 12-week-old mice were used for morphometry and immunostaining. For histological analysis, pancreases were fixed in 10% formalin, paraffin-embedded, and sectioned in 6 μm (each section spaced at least 50 μm apart), 4 sections of each animal were used. Pancreas sections were stained with hematoxylin and eosin for histological study. To measure islet area, sections were photographed at low magnification. Images were imported to a software (ImageJ, NIH, Bethesda, MA, USA), and total pancreas and islet areas were selected. Pixel numbers in selected areas were used to calculate the relative islet area to total pancreas.

### Glucose tolerance test

Mice were subjected to glucose tolerance test at 8-10 weeks of age. Mice were fasted overnight (16 hours) and intraperitoneally injected with 0.9% NaCl solution containing D-glucose at the dose of 1 or 2g/kg body weight. Blood samples for glucose measurements were taken at 0, 5, 10, 15, 30, 60 and 120 minutes after the glucose load. Serum was also collected at 0, 5, 30 minutes after glucose load, insulin concentration was detected using mouse insulin ELISA Kit (Crystal Chem Inc., Chicago, Illinois, USA)

### Multiple low-dose streptozotocin (MLD-STZ) treatment

Mice were intraperitoneal injected with STZ (Sigma Chemicals, St Louis, MO, USA) of 40 mg/kg body weight for five consecutive days. Blood glucose levels were measured twice a week using a glucometer (FreeStyle, Abbott, Alameda, CA, USA). Mice were considered diabetic when blood glucose levels were > 200 mg/dl in two consecutive measurements. Mice were killed on day 25 of the study; pancreases and serum were collected and used for further evaluation.

### Cell culture and RNA interference

Min-6 cells were cultured in 25mmol/l glucose Dulbecco's modified Eagle's medium (DMEM), supplemented with 15% fetal bovine serum, 50 μM β-mercaptoethanol, 100 units/ml penicillin, 100 μg/ml streptomycin, and 2 mmol/l l-glutamine at 37°C, 5% CO_2_. Cells were trypsinized, counted, and plated in culture medium without antibiotics at a density of ~ 40-50%, 24 hours before transfection. The on-target HDAC3 and Suppressors of cytokine signaling 3 (SOCS3) siRNAs (Thermo Scientific, Lafayette, CO, USA) as well as non-targeting sequence siRNA were transfected at a working concentration of 50 nM using Lipofectamine RNAiMax (Invitrogen, Carlsbad, CA, USA) according to the manufacturer's instruction. Transfected cells were cultured for 72 hrs for glucose-stimulated insulin secretion, flowcytometry analysis and Real-time PCR.

### RNA isolation and quantitative real-time PCR

Total RNAs were isolated using Qiazol (Qiagen, Valencia, CA, USA) and subjected to reverse transcription using High Capacity cDNA Reverse Transcription Kit ((Applied Biosystems, Foster City, CA, USA). Real-time PCR was performed in triplicates using FastStart Universal SYBR Green Master (Roche Diagnostics Corp., Indiana, USA) on ABI 7900 HT Real-Time PCR system (Applied Biosystems, Foster City, CA, USA). Data were normalized to mouse β-actin mRNA.

### Chromatin immunoprecipitation (CHIP) assay

CHIP assays were performed using an EZ-CHIP kit (Millipore, Billerica, MA, USA), following the manufacturer's instructions. CHIP-grade anti-HDAC3 (Abcam, Cambridge, MA, USA), rabbit IgG negative control antibody (eBioscience, San Diego, CA, USA) and anti-RNA polymerase II (Millipore, Billerica, MA, USA) were used to immunoprecipitate the protein-DNA complexes. Purified immunoprecipitated DNA was analyzed by PCR with primers for SOCS3 promoter (Table S1).

### Glucose-stimulated insulin secretion and insulin content test

The cultured cells were washed and pre-incubated for 60 min in Krebs-Ringer bicarbonate buffer (129.4 mmol/l NaCl, 5.2 mmol/l KCl, 1.3 mmol/l KH_2_PO_4_, 2.7 mmol/l CaCl_2_, 1.3 mmol/l MgSO_4_, 24.8 mmol/l NaHCO_3_, 10 mmol/l HEPES, 0.1% BSA [pH 7.^4^]) with 2.8 mmol/L glucose. Upon completion of the incubation, the buffer was removed completely and replaced with fresh KRB containing 20 mmol/L glucose for an additional 45 min. The supernatant was collected for measuring insulin secretion.

The total insulin in the cells as well as the whole pancreas was extracted by acid ethanol (0.18 M HCl in 95% ethanol) treatment overnight. Insulin concentration was measured using mouse insulin ELISA Kit (Crystal Chem Inc., Chicago, Illinois, USA). Insulin secretion and content were normalized to total protein.

### Flow cytometry (FACS)

Min-6 cells were trypsinized, washed twice with FACS staining buffer (PBS, 3% FBS), and then incubated with Fc block (clone 2.4G2). Trypsinized cells were first fixed and permeabilized by Foxp3 Fixation Buffer and Permeabilization Buffer (eBioscience), and then incubated with Fc blocker (clone 24G2). Treated cells were then stained with related antibodies: anti-HDAC3 (ab7030, Abcam), anti-SOCS3 (ab16030, Abcam), or rabbit IgG as isotype control, followed by incubation with FITC or PE-conjugated anti-rabbit IgG (eBioscience, 1:100 dilution). Data was harvested by FACSAriaII Flow Cytometer (BD Biosciences) and analyzed using FlowJo software (FlowJo, LLC).

### Statistical analysis

Statistical analysis was performed with Prism 5.0 (GraphPad Software, San Diego, CA). A two-tailed Student t test was used to test the differences between two groups. ANOVA was used to compare the means of multiple groups. Data are presented as mean ± standard deviation (SD). Gehan-Breslow-Wilcoxon test was used for the analysis of the incidence of diabetes. Differences were considered significant with a *p* value < 0.05.

## SUPPLEMENTARY MATERIAL TABLE


