# Supplementary material for: Conditional ablation of HDAC3 in islet beta cells results in glucose intolerance and enhanced susceptibility to STZ-induced diabetes
Source: Oncotarget. 2016 Aug 15;7(36):57485–97. doi: 10.18632/oncotarget.11295 (PMC5295367; doi:10.18632/oncotarget.11295)
Supplement: Supplementary file 1 [file oncotarget-07-57485-s001.pdf]

Conditional ablation of HDAC3 in islet beta cells results in glucose intolerance and enhanced susceptibility to STZ-induced diabetes

Supplementary Material

Methods

Islet isolation, dispersion, immunofluorescent staining, and pancreas histological and immunofluorescence Analysis

Mouse islets were isolated by type V collagenase (Sigma Chemicals, St Louis, MO, USA) digestion of pancreas. Isolated islets were exposed to 0.25% trypsin-EDTA at 37°C for 10 min, followed by gently pipetting for 1-2 min to dissociate into single cells. Dispersed islets cells were fixed in 4% (vol./vol.) paraformaldehyde for 10 min, permeabilized with 0.25% (vol./vol.) Triton X-100 for 10 min and blocked overnight in 1% (wt/vol.) BSA at 4°C. Cells were then labeled overnight at 4°C with polyclonal anti-HDAC3 (Abcam, Cambridge, MA, USA) and monoclonal anti-insulin ((Linco, Research, Inc., St Louis, MO, USA). Cells were then stained at room temperature in dark for 60 min with PE-conjugated goat anti-rabbit, FITC-conjugated goat anti-mouse secondary antibodies (eBioscience, San Diego, CA, USA) respectively. Cells were washed and mounted on glass slides which were viewed on a microscope (Leica Microsystems, Wetzlar, Germany).

Pancreases obtained from 8- to 12-week-old mice were used for morphometry and immunostaining. For histological analysis, pancreases were fixed in 10% formalin, paraffin-embedded, and sectioned in 6 μm (each section spaced at least 50 μm apart), 4 sections of each animal were used. Pancreas sections were stained with hematoxylin and eosin for histological

study. To measure islet area, sections were photographed at low magnification. Images were imported to a software (ImageJ, NIH, Bethesda, MA, USA), and total pancreas and islet areas were selected. Pixel numbers in selected areas were used to calculate the relative islet area to total pancreas.

Table S1. PCR primer sequences

Pirmer Name	Sequences
HDAC3 $^{flox/flox}$	Forward 5’-GGACACAGTCATGACCCGGTC-3’<br>Reverse 5’-CTCTGGCTTCTGCTATGTCAATG-3’
Rip-Cre	Forward 5’-CTCTGGCCATCTGCTGATCC-3’<br>Reverse 5’-CGCCGCATAACCAGTGAAAC-3’
Internal Control	Forward 5’-CAAATGTTGCTTGTCTGGTG-3’<br>Reverse 5’-GTCAGTCGAGTGCACAGTTT-3’
SOCS3 promoter	Forward 5’-GCTGAATGGTCCTACGTCCCTT-3’<br>Reverse 5’-TACAGTTCCAAGCATCCCGTG-3’
